# Mechanisms of Colorectal Cancer Prevention by Aspirin—A Literature Review and Perspective on the Role of COX-Dependent and -Independent Pathways

**DOI:** 10.3390/ijms21239018

**Published:** 2020-11-27

**Authors:** Ranjini Sankaranarayanan, D. Ramesh Kumar, Meric A. Altinoz, G. Jayarama Bhat

**Affiliations:** 1 Department of Pharmaceutical Sciences and Translational Cancer Research Center, College of Pharmacy and Allied Health Professions, South Dakota State University, Brookings, SD 57007, USA; ranjini.sankaranarayanan@sdstate.edu; 2 Department of Entomology, University of Kentucky, Lexington, KY 40506, USA; rku233@uky.edu; 3 Department of Biochemistry, Acibadem M.A.A. University, Istanbul, Turkey; maltinoz@gmail.com

**Keywords:** colorectal cancer, aspirin, salicylic acid, hydroxybenzoic acid, gentisic acid, 2,5-dihydroxybenzoic acid, 2,3-dihydroxybenzoic acid, gut microbiota, cyclooxygenase, cyclin dependent kinases

## Abstract

Aspirin, synthesized and marketed in 1897 by Bayer, is one of the most widely used drugs in the world. It has a well-recognized role in decreasing inflammation, pain and fever, and in the prevention of thrombotic cardiovascular diseases. Its anti-inflammatory and cardio-protective actions have been well studied and occur through inhibition of cyclooxygenases (COX). Interestingly, a vast amount of epidemiological, preclinical and clinical studies have revealed aspirin as a promising chemopreventive agent, particularly against colorectal cancers (CRC); however, the primary mechanism by which it decreases the occurrences of CRC has still not been established. Numerous mechanisms have been proposed for aspirin’s chemopreventive properties among which the inhibition of COX enzymes has been widely discussed. Despite the wide attention COX-inhibition has received as the most probable mechanism of cancer prevention by aspirin, it is clear that aspirin targets many other proteins and pathways, suggesting that these extra-COX targets may also be equally important in preventing CRC. In this review, we discuss the COX-dependent and -independent pathways described in literature for aspirin’s anti-cancer effects and highlight the strengths and limitations of the proposed mechanisms. Additionally, we emphasize the potential role of the metabolites of aspirin and salicylic acid (generated in the gut through microbial biotransformation) in contributing to aspirin’s chemopreventive actions. We suggest that the preferential chemopreventive effect of aspirin against CRC may be related to direct exposure of aspirin/salicylic acid or its metabolites to the colorectal tissues. Future investigations should shed light on the role of aspirin, its metabolites and the role of the gut microbiota in cancer prevention against CRC.

## 1. Introduction

Colorectal cancer (CRC) is the second most common cause of cancer related deaths [1,2] and the third most common cancer diagnosis [3] in the United States of America. Every year in the United States, around 150,000 cases of CRC are predicted and 1/3 of the affected patients die from this disease. Colorectal cancer usually begins as a benign adenomatous polyp that develops into an advanced form of adenoma with high-grade dysplasia, which eventually leads to the development of invasive cancer [4]. Formation of benign precancerous polyps that is characterized by the aggregation of abnormally dividing cells in the intestinal lumen marks the initiation of CRC. Over time, these polyps gain numerous mutations and acquire the ability to invade the bowel wall and eventually spread to the adjacent lymph nodes, and distant metastatic sites. The risk of a polyp developing into CRC increases with increasing size of the polyps owing to increased genetic changes and accumulation of epigenetic factors. As genetic damage increases over time, features of high-grade dysplasia start to present, and if left untreated, the polyps will develop the ability to invade adjacent tissues and grow beyond the confinement of the colon and rectum [4].

Aspirin and other non-steroidal anti-inflammatory drugs (NSAIDs) have shown promising anti-cancer effects upon regular consumption for 5 or more years [5,6,7,8]. The first report linking aspirin use and reduction in CRC was published in 1988 by Kune et al. that showed a statistically significant reduction in cases taking aspirin-containing medication in both men and women [9]. Numerous epidemiological and clinical studies thereafter proved aspirins’ efficacy to reduce CRC. These include (1) observational case-control studies [9,10], (2) randomized control trials in subjects with sporadic colorectal adenomas [11], (3) randomized control trials in subjects with Lynch syndrome [12,13] and (4) individual patient data meta-analysis of random control trials in the prevention of vascular events [14]. These studies indicated a decrease in the occurrence of CRC upon aspirin consumption and the overall incidences of gastrointestinal (GI) cancers in individual cases highly correlated with that of randomized trials [10,14]. It was also suggested that daily use of aspirin was associated with a significant reduction in colorectal adenomas in patients with previous incidences of CRC [11]. The reduction in CRC reported in these studies varied between 20 and 40% [8,15,16]. Aspirin was also shown to decrease chemically induced carcinogenesis in colorectal tissues using animal models [17,18,19,20]. Interestingly, data from epidemiological and clinical studies indicated that low-dose aspirin (75–81 mg) is as effective in preventing CRC as higher doses (≥325 mg) [7,21]. Aspirin consumption has been shown to cause GI bleeding and renal toxicity in some individuals, with an incidence of less than 4 per 1000 cases [22,23]. However, due to the increasing body of evidence for aspirin’s efficacy against CRC, it is argued that the benefits outweigh the risk. Hence, the United States Preventive Services Task Force (USPSTF), recommended “initiating low dose aspirin use for the primary prevention of cardiovascular disease and colorectal cancer in adults aged 50–59 years” in 2016 [24]. Following this, numerous clinical trials have been launched to establish its clinical use against CRC [25,26,27,28].

An intriguing aspect of aspirin against CRC is its dose independent effect [29]. Multiple mechanisms in cells have been proposed for aspirin’s anticancer effects and this includes inhibition of enzymes, modulation of signaling pathways and transcription factors, and post translational modifications of proteins, although a consensus has not been reached to date. The intent of this review is to discuss some of these potential mechanisms reported in the literature and assess their strengths and limitations in light of recently published papers.

## 2. Pharmacological Effects of Aspirin

Following oral consumption, aspirin is absorbed mainly in the stomach and upper small intestine [30]; in addition, lymphatic uptake of aspirin has also been recently reported [31]. Aspirin concentration maximally achieved in the plasma varies depending on the dose with anti-platelet (75 mg/day), analgesic (325–600 mg/4–6 h) and anti-inflammatory (1.2 g/4–6 h) doses reaching 7.31 μM, 25–80 μM, and 144 μM, respectively [16]. The half-life of aspirin is ~20 min and it undergoes hydrolysis by intestinal, plasma and liver esterases to generate salicylic acid [16,30]. The concentration of salicylic acid in the plasma has been estimated to be around 15 μM for anti-platelet doses, 500 μM for analgesic doses and 1.5–2.5 mM for anti-inflammatory doses. The half-life of salicylic acid is estimated to be 4–6 h in the plasma [16]. It is reported that the oral bioavailability of aspirin tablets is ~40–50%; however, some reports have estimated that aspirin’s oral bioavailability is ~68% [32]. It is important to note that the oral bioavailability of aspirin is significantly reduced in enteric coated and sustained release tablets [33].

Aspirin like all other NSAIDs works through the inhibition of cyclooxygenase (COX; COX-1 and COX-2) enzymes in the body. COX-1 is the only isoform present in mature platelets and is responsible for platelet aggregation. COX-2 is not expressed under normal conditions; however, it is expressed in many tissues during inflammation, wound healing, and neoplasia [29,34]. The molecular mechanism of aspirin involves its ability to inhibit arachidonic acid metabolism through prostaglandin H (PGH) synthase or the COX pathway. Aspirin inhibits COX-1 by acetylating Ser530, and acetylates COX-2 at Ser516, thereby inactivating them [35,36,37]. Depending on the cell type, inhibition of COX activity affects synthesis of different prostaglandins (PGs) including PGD2, PGE2 and PGF2α, prostacyclin (PGI2) and thromboxane A2 (TXA2). TXA2 is the major metabolite that promotes the activation and aggregation of platelets [38]. It is to be noted that aspirin is more selective to COX-1 as compared to COX-2, with IC_50_ for COX-1 inhibition observed at 1.7 μM compared to the >100 μM required for COX-2 inhibition [39,40].

## 3. Mechanisms Proposed for Aspirin’s Chemopreventive Effects

As mentioned previously, daily use of aspirin has been shown to reduce the risk of CRC and recurrence of adenomatous polyps. This observation sparked the interest of scientists, physicians and the public alike for its potential use as a prophylactic drug against CRC and studies conducted based on this interest have led to numerous hypotheses on its mechanism of action. Some of the major pathways proposed for its anti-cancer effects are discussed below.

### 3.1. Inhibition of COX Enzymes: The Platelet Hypothesis

Cyclooxygenases are a group of enzymes that are involved in the synthesis of prostanoids including PGs and TXA2 from arachidonic acid. While TXA2 is majorly involved in platelet aggregation, prostaglandins such as PGE2 have a role in inflammatory reactions. Of the two COX enzymes, COX-2 overexpression is associated with cancer progression. Unlike COX-1 that is constitutively present in all cell types including platelets, COX-2 is absent under normal conditions in many cell types. However, as mentioned above, COX-2 expression is elevated under inflammatory and other pathological conditions such as cancer [29,34]. Owing to the involvement of COX-2 in cancer [41] it is suggested that aspirin’s anti-cancer effects may occur through the inhibition of this enzyme. Additionally, studies conducted using COX-1 and COX-2 deletions have also demonstrated reduced intestinal tumorigenesis [42]. However, a direct inhibition of COX-2 by low-dose aspirin is thought to be unlikely due to the observed low plasma concentration (~7 μM) and a requirement for higher amounts for enzyme inhibition (IC_50_ 278 μM) [40]. Thus, the fact that low-dose aspirin is as effective as higher doses in preventing CRC led to the suggestion that COX-1 inhibition in platelets could indirectly inhibit COX-2 expression in the colorectal tissues through sequential steps [6,16,29].

**Figure 1:**
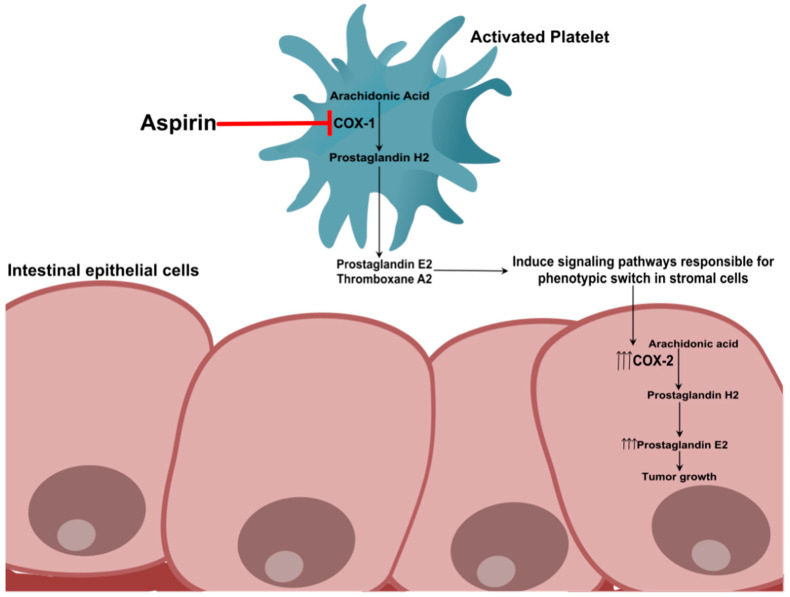
“Platelet hypothesis”. According to this model, platelet activation and aggregation at the site of intestinal mucosal injury is fundamental to tumorigenesis in the colorectal tissues. Activation of platelets can lead to the release of lipid mediators (e.g., prostaglandin (PG)E2) and growth factors (e.g., platelet derived growth factors (PDGF)) from platelets that in turn may promote stromal cell growth and induce cyclooxygenases (COX)-2 expression in intestinal cells of the colonic mucosa. Such events can further lead to increased PGE2 production resulting in hyperplasia, epithelial–mesenchymal transition (EMT) and transformation to a neoplastic phenotype [6,29]. It is hypothesized that central to the prevention of colorectal cancer (CRC) by aspirin is its ability to prevent platelet aggregation through inhibition of COX-1 leading to subsequent inhibition of thromboxane (TX)A2. Prevention of platelet aggregation blocks the release of lipid mediators and growth factors from platelets that otherwise induces the expression of COX-2 (in epithelial cells), which is implicated in tumorigenesis. This hypothesis thus states that sequential inhibition of COX-1 in platelets followed by inhibition of COX-2 expression in adjacent nucleated cells of the colorectal mucosa are important steps in aspirin’s cancer preventive actions.

According to this theory (popularly referred to as the “platelet hypothesis” [29]) the initiating event for tumorigenesis in the colorectal tissues is the aggregation and activation of platelets in the intestinal mucosa upon injury. Persistent activation of platelets, if left unchecked, will result in the local recruitment of immune cells, leading to inflammation. Aggregation of platelets at these sites release platelet-derived growth factors (PDGF), cytokines (interleukin-1β) and lipid mediators (PGE2 and TXA2) that then promote growth/proliferation of adjacent nucleated cells in the colonic mucosa. It is further hypothesized that these agents may then increase the levels of COX-2 (further increasing PGE2 levels) and result in epithelial–mesenchymal transition (EMT) of the intestinal epithelial cells, characteristic of early events in tumorigenesis. EMT activation in turn results in the generation of tumor-initiating cells and triggers tumor cell invasion and metastasis to distant organs [6,16,29]. It is to be noted that, PGE2 binding to EP1-4 receptors (G-protein coupled receptors) activates signal transduction pathways that promote adhesive, migratory and invasive behavior of cells during the development and progression of cancer [43]. Therefore, according to the platelet hypothesis, platelet aggregation and aberrant activation at site of mucosal injury in the GI tract is the driving factor for the genesis of adenomatous polyps and eventual progression to CRC. Aspirin’s chemopreventive effect against CRC is thus attributed to its ability to inhibit COX-1 in platelets, prevent the release of lipid or protein mediators of inflammation and subsequently decrease COX-2 expression in colorectal tissues (Figure 1).

### 3.2. Inhibition of Cyclin Dependent Kinases (CDKs) by Aspirin Metabolites: The Metabolite Hypothesis

**Figure 2:**
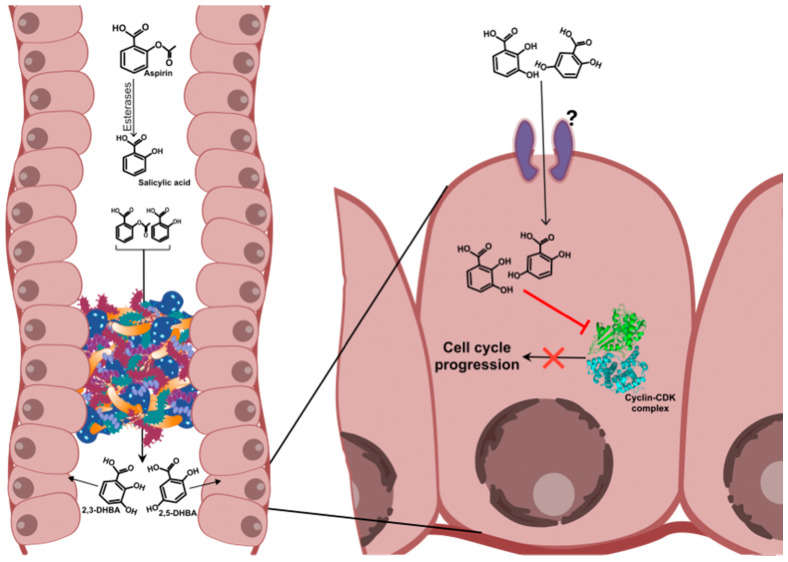
Metabolite hypothesis. According to this hypothesis, the unabsorbed aspirin and salicylic acid in the intestine and colon are biotransformed by the colonic microbiota to generate metabolites such as 2-3-dihydroxybenzoic acid (2,3-DHBA) and 2,5-DHBA. These HBAs are taken up by the colonic epithelial cells and may arrest cell growth by inhibiting cyclin dependent kinases (CDKs) and/or other proteins. It is suggested that a reduced rate of cell proliferation would provide the cells with an opportunity to repair damaged DNA, enhancing genetic stability. Alternatively, it would also provide cells of the immune system time to recognize tumor cells through immune surveillance and destroy them [44].

According to this recently proposed hypothesis, metabolites of aspirin/salicylic acid (2-3-dihydroxybenzoic acid (2,3-DHBA/pyrocatechuic acid) and 2,5-dihydroxybenzoic acid (2,5-DHBA/gentisic acid)) are key contributors to its anti-cancer effects against CRC (Figure 2) [44]. This is supported by the observations in our laboratory that have demonstrated that both 2,3-DHBA and 2,5-DHBA are capable of inhibiting cancer cell growth in vitro [45]. While 2,5-DHBA was universally effective in arresting cancer cell growth in colon cancer cell lines (HCT-116 and HT-29) and breast cancer cell lines (MDA-MB-231), 2,3-DHBA was more selective against MDA-MB-231 cell line; the concentration of the metabolites required to inhibit colony formation varied with each cell type. In HT-29 and HCT-116 cells, the inhibition with 2,5-DHBA was observed between 250 and 500 μM while in MDA-MB-231 cells, it required 50–100 μM of 2,5-DHBA. In contrast, 2,3-DHBA showed effective inhibition at ~500 μM in MDA-MB-231 cells. Our studies also indicated that CDKs are likely to be cellular targets of 2,3-DHBA and 2,5-DHBA. Both 2,3-DHBA and 2,5-DHBA were capable of inhibiting CDKs, particularly CDK1 and CDK6 [45,46].

It is hypothesized that 2,3-DHBA and 2,5-DHBA are generated in the body due to microbial transformation of aspirin and salicylic acid that are left unabsorbed in the intestine. Previous studies have reported that only 40–50% of the orally consumed aspirin is bioavailable [16], and that aspirin undergoes rapid hydrolysis in the intestine to generate salicylic acid [32]. With enteric coated tablets, the bioavailability of aspirin is further reduced [16,33] and therefore more aspirin may be left unabsorbed in the intestine. Aspirin and salicylic acid remaining in the intestine may then be subjected to biotransformation by the resident microbiota. In support of this, Kim et al. demonstrated the conversion of aspirin and salicylic acid to hydroxy salicylic acids, mediated by the human gut microflora. Additionally, when rats were administered with ampicillin and aspirin, these authors showed that the aspirin metabolizing activity by the bacteria was significantly reduced as evidenced from fecal samples, and the levels of aspirin in the plasma doubled [47]. In a recent report it was also demonstrated that administration of aspirin to rats along with amoxicillin decreased aspirin metabolism in the intestine as compared to treatment with aspirin alone [48]. These two reports suggest that the biotransformation of aspirin by the gut microflora may be a source of 2,3-DHBA and 2,5-DHBA. Although aspirin and salicylic acid are also metabolized in the liver to generate 2,3-DHBA and 2,5-DHBA, they are reported to be minor metabolites of liver biotransformation [49]. Therefore, it is unlikely that these liver generated DHBAs, with low concentrations in the plasma, contribute to the anti-cancer effects of low-dose aspirin on colorectal tissues.

The in vitro studies which demonstrated the inhibitory effect of 2,3-DHBA and 2,5-DHBA on colon cancer cell growth suggested that the metabolites are effective at a concentration ranging from 100–500 μM [45]. We believe that these concentrations are pharmacologically achievable in the human intestine. It is reported that the intestinal fluid volume ranges between 160 and 750 mL depending on fasting or fed conditions respectively [50]. Considering that 50% of the aspirin/enteric-coated aspirin remains in the intestine/colon after oral ingestion, a dose of 81 mg aspirin will reach concentrations between 0.3 and 1.4 mM depending on the intestinal fluid volume. Hence, the concentration of 2,3-DHBA and 2,5-DHBA required to exert anti-cancer effects would be achievable from the biotransformation of aspirin remaining in the intestine.

Interestingly, it should be noted that 2,5-DHBA can also be produced through the oxidation of salicylic acid in human body fluids [51]. Additionally, it has been reported that other therapeutic drugs such as 5-aminosalicylic acid (5-ASA) can generate this HBA through oxidation in the presence of hypochlorous acid released from activated leukocytes during colonic inflammation [52]. Studies have shown a decreased risk for CRC in ulcerative colitis patients treated with 5-ASA [53], and therefore it is possible that the mechanism may involve the formation of 2,5-DHBA. These examples are indicative of the possibility of 2,5-DHBA being generated in the vicinity of precancerous fields of the colonic mucosa where elevated pro-inflammatory and pro-oxidant features are observed.

Besides our work, a few other reports published in the literature substantiate a role for aspirin metabolites in cancer prevention. One report demonstrated the ability of 2,5-DHBA to enhance survival in Ehrlich breast carcinoma-bearing mice following oral administration. This study reported that 2,5-DHBA was highly effective in inhibiting cell proliferation in C6 rat glioblastoma cells at various concentrations ranging from 2 to 16 μg/mL [54]. With regard to targets, it will be interesting to determine if 2,5-DHBA has other cellular targets, besides CDKs. For example, in a previous study, it was demonstrated that 2,5-DHBA can inhibit fibroblast growth factor (FGF) receptor function suggesting that this pathway may also contribute to aspirin’s anti-cancer effects [55]. 2,5-DHBA has also been shown to inhibit COX-2 mediated PGE2 synthesis in murine macrophages [56]. Interestingly, 2,5-DHBA is implicated in the inhibition of COX enzymes through its antioxidant properties [57]. A recent study also showed that conjugation of 2,5-DHBA with gelatin induces heparin-like characteristics and inhibited migration and invasion of cancer cells [58]. As 2,5-DHBA potently inhibits growth of cancer cells [45,54,59], it is possible that it may alter the structure of matrix proteins surrounding cancer cells and inhibit their invasive potential. Altogether, these examples suggest that 2,5-DHBA may be an important metabolite responsible for aspirin’s chemopreventive actions. A detailed account of the potential role of 2,5-DHBA and its targets has been published in a separate review [59].

Interestingly, Hydroxybenzoic acids (HBAs) are also generated from flavonoid degradation and are also present in fruits and vegetables [60,61,62]. The metabolite hypothesis thus proposes that HBAs generated from the degradation of aspirin/salicylic acid, flavonoids and those HBAs present in the diet may contribute to their chemopreventive properties [44]. This attractive theory requires further validation and in vivo experiments using germ free mice, which would provide information on the role of HBAs in aspirin’s anti-cancer effects against CRC. Additionally, a quantitative estimation of these metabolites generated in the intestine from aspirin/flavonoid degradation will establish whether pharmacologically relevant concentrations of HBAs are achievable in situ.

### 3.3. Inhibition of Nuclear Factor (NF)-κB Signalling

NF-κB is a transcription factor that is involved in many immune and inflammatory responses [63]. NF-κB is present as a heterodimer along with IκB in the cytoplasm. Upon receiving appropriate signals, IκB is phosphorylated by IκB Kinase (IKK) that eventually leads to the ubiquitin mediated degradation of IκB. The free NF-κB then translocates to the nucleus where it acts as a transcription factor for many cellular processes including those that control cellular growth. Aspirin and salicylic acid have been shown to inhibit IKK, through direct interactions, thus preventing the translocation of NF-κB to the nucleus. As aspirin is also an NSAID, it is additionally believed to prevent inflammation mediated carcinogenesis by affecting this signaling pathway. The effect exerted by aspirin on NF-κB requires concentrations ranging from 0.05 to 5 mM [63,64].

### 3.4. Activation of AMP-Kinase and Inhibition of mTOR Signaling

Adenosine monophosphate activated protein kinase (AMPK) is a critical cellular energy sensor that gets activated in response to stress. AMPK is involved in processes that synthesize ATP to meet the energy demands of the body. AMPK regulates numerous pathways including that of p53, fatty acid synthase, and mechanistic target of rapamycin (mTOR). As these pathways are crucial for cell survival and metabolism, AMPK has also been implicated in cancer progression in numerous cancers including CRC [65]. It has been shown that aspirin, at a concentration of 5 mM is capable of decreasing mTOR signaling by inhibiting the effectors S6K1 and 4E-BP1, that are involved in translation and protein synthesis, eventually leading to cell death. It was also demonstrated that aspirin could alter the AMP:ATP ratios in cells, leading to the activation of AMPK, and subsequent inactivation of mTOR signaling. Aspirin also inhibited mTOR signaling in CRC patients treated with analgesic doses (600 mg/day for one week). In these patients, there was a decreased phosphorylation of S6K1 and S6 effectors, suggesting that their inhibition helps in the regulation of intracellular energy homeostasis and metabolism, contributing to the protective effect of aspirin against CRC [66].

### 3.5. Inhibition of Wnt Signalling and β-Catenin Phosphorylation

Wnt-signaling is constitutively activated in progenitor cells of the colorectal epithelium, whereas in mature/maturing cells this pathway is inactivated by the expression of adenomatous polyposis coli (APC). APC acts by binding to the excess β-catenin in the cytoplasm, leading to the subsequent ubiquitination and proteolysis of this onco-protein. Initiation of CRC development is marked by aberrant activation of the Wnt-signaling pathway that is characterized by the mutation of the APC gene, rendering it non-functional [67]. This mutation in the tumor suppressor gene tips the balance to favor uncontrolled proliferation and differentiation of the colonic epithelia. It has been demonstrated that aspirin, tested at concentrations between 0.05 and 5 mM, stimulates the phosphorylation (through inhibition of protein phosphatase 2A) and subsequent ubiquitination of β-catenin, leading to the inhibition of the Wnt/β–catenin pathway [68].

### 3.6. Downregulation of c-Myc, Cyclin A2 and CDK2

c-myc is a nuclear transcription factor that controls 15% of the expression of all genes. It regulates multiple cellular functions such as cell proliferation, metabolism, apoptosis, growth and differentiation. It is frequently overexpressed in nearly 20% of all cancers, and this is associated with poor clinical outcome and an aggressive metastatic phenotype. Aspirin/salicylic acid has been shown to downregulate the levels of c-myc mRNA and protein, between 0.25 and 2.5 mM, in a variety of cancer cells [69], and similar results have been reported in another study [70]. Aspirin and salicylic acid have also been shown to downregulate cell cycle regulatory proteins cyclin A2 and CDK2 through proteasomal mediated pathways at concentrations ranging from 0.5 to 2.5 mM [71]. It is suggested that this may lead to cell cycle arrest and inhibition of cell proliferation.

### 3.7. Induction of Polyamine Catabolism

Ornithine decarboxylase (ODC) is the first enzyme involved in polyamine synthesis. The transcriptional regulator c-myc activates the transcription of ODC which results in increased levels of mucosal polyamines that is implicated in tumorigenesis. Aspirin use, between 20 and 100 μM, in Caco-2 cells has been associated with the transcriptional activation of spermidine/spermine N1-acetyltransferase (SSAT), a driver for polyamine catabolism. Reduced levels of polyamines are thus believed to result in reduced tumorigenesis [72].

### 3.8. Induction of DNA Mismatch Repair Proteins

DNA mismatch repair is a mechanism that is evolutionarily conserved, to repair mutations that arise during DNA replication or damage. Epigenetic or somatic changes to mismatch repair genes have been associated with CRC development [73]. It was demonstrated that in colon cancer cells, exposure of aspirin between concentrations of 1 to 10 mM induced DNA mismatch repair proteins by 2–7-fold, depending on the cell line studied. This in turn was associated with G0/G1 cell cycle arrest and apoptosis. The authors suggested that this COX-independent pathway may contribute to aspirin’s anticancer effects [74].

### 3.9. Acetylation of p53, Glucose-6-Phosphate Dehydrogenase and Other Proteins

p53 is a tumor suppressor protein that regulates cell proliferation. In normal cells, it is activated through acetylation and phosphorylation following which it translocates to the nucleus and acts as a transcription factor. Nearly 50% of all tumors contain a mutated p53 and such mutations inactivate its tumor suppressor functions leading to enhanced cell proliferation. Aspirin has been shown to directly acetylate both wild-type and mutant p53, at concentrations between 0.05 and 2.5 mM, and this was associated with induction of p21 and Bax, suggestive of a role in restoration of p53 function and tumor suppression [75,76,77]. Aspirin was also shown to acetylate glucose-6-phosphate dehydrogenase (G6PD; at ≥100 μM), an enzyme in the hexose monophosphate shunt pathway and cause decreased enzyme activity. It was suggested that selective inhibition of G6PD may represent an important mechanism by which aspirin may exert its anticancer effects through inhibition of ribonucleotide synthesis [78,79]. Aspirin’s ability to decrease G6PD, potentially contributing to prevention of cancer cell growth, is an interesting finding because there are reports that have documented a direct correlation between G6PD deficiency and decreased cancer incidences [80].

Aspirin also appears to acetylate multiple proteins, over a range of concentrations starting from 100 μM, in cells grown in culture as well as in vivo [76,78,81,82]. Two independent reports have documented aspirin’s ability to acetylate multiple proteins which include histones, cytoskeletal and heat-shock proteins, glycolytic and pentose pathway enzymes, proteins involved in translation, proteasomal subunits and mitochondrial proteins. The significance of these post-translational modifications has not been thoroughly investigated [78,81]. Interestingly, Tatham et al. reported that although exposure of cells to aspirin amplifies the acetylation status of proteins by several orders of magnitude, protein function is not altered as long as deacetylases are not inhibited. They suggested that aspirin-mediated acetylations do not possibly accumulate to levels capable of eliciting biological effects in most cases [82].

### 3.10. Other Mechanisms

In addition to the pathways mentioned above, aspirin is also known to affect other targets. For example, it is suggested that aspirin (between 1–4 mM) might exert its chemopreventive effect against CRC by normalizing epidermal growth factor receptor expression in gastrointestinal precancerous lesions [83]. It is also reported that at concentrations between 0.1 and 5 mM, aspirin and salicylic acid could inhibit 12-*O*-tetradecanoylphorbol-13-acetate (TPA) induced activator protein 1 activity, preventing neoplastic transformation [84]. Another study showed that aspirin’s ability to inhibit colon cancer cell growth (between 5 and 10 mM) may involve downregulation of specificity protein transcription factors [85]. Both aspirin and salicylate are also reported to induce apoptosis through caspase activation in B-cell chronic lymphocytic leukemia cells at a concentration of 10 mM [86]. In addition, aspirin has been shown to exert cytotoxic and anti-cancer effects through generation of reactive oxygen species in melanoma cells at concentrations between 0.1 and 1 mM [87]. A recent study has also demonstrated that low-dose aspirin (100 mg/day for 7 days) causes acetylation of COX-1, inhibiting PGE2 synthesis, in human intestinal mucosal cells leading to decreased levels of S6 kinase, suggesting that this pathway may play a role in the prevention of colorectal carcinogenesis [88].

**Figure 3:**
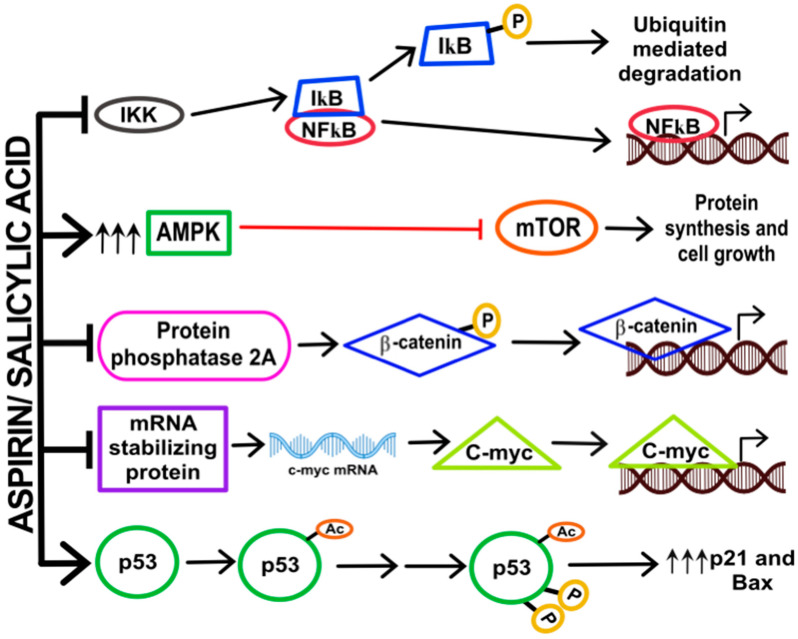
Other pathways. Aspirin/Salicylic has been shown to modulate a variety of signaling pathways which may also contribute to its anti-cancer effects. It is known to inhibit nuclear factor (NF)-κB pathway, activate the adenosine monophosphate activated protein kinase (AMPK) pathway, inhibit the Wnt/β-catenin signaling pathway, cause degradation of c-myc mRNA and proteins, and acetylate p53 leading to induction of p21 and Bax protein expression, to name a few. See text for details; additional pathways described in the text are not depicted in this figure. -P, phosphorylated protein; -Ac, acetylated protein.

It is important to note that most of the proposed mechanisms of cancer prevention by aspirin were studied in cancer cell lines, and therefore our current understanding of these pathways comes mainly from in vitro studies. Therefore, additional in vivo studies in greater depth are required to confirm these in vitro findings and fully establish the exact mechanisms of CRC prevention by aspirin. A compilation of some of these pathways is diagrammatically represented in Figure 3.

## 4. Potential Role of the Gut Microbiota in Aspirin’s Effect against CRC

In many studies, variability has been reported in aspirin’s ability to decrease the occurrences of cancers. While genetic reasons may account for some of this variability, it may also depend on the levels of HBAs generated in the intestine though cytochrome P450 (CYP450)-mediated metabolism within the cells or microbial degradation in the lumen. In this regard, a report showed that in patients carrying a CYP2C9 variant allele, there was a decreased risk reduction in colorectal adenomas, following aspirin administration. They suggested that this may be because of the inability of CYP2C9 to generate 2,5-DHBA [89], which again highlights the importance of aspirin metabolites in cancer prevention. On the other hand, microorganisms may also significantly differ with respect to their ability to biotransform aspirin and salicylic acid to generate HBAs. The intestinal microbiota may vary depending on the diet and therefore both diet and microbial composition in the intestine may play a role in HBA generation, accounting for the observed variability in epidemiological studies. Several reports have documented that diet influences the composition of the microbiota [90,91,92] and if HBAs are indeed important contributors to the cancer preventive properties of aspirin, newer strategies are required to achieve the desired chemopreventive effects in all population. This may include adjusting to a healthier diet that incorporates more fruits and vegetables along with aspirin, or consumption of appropriate probiotics along with aspirin in populations where dietary adjustment is difficult.

Interestingly, in one randomized clinical study, aspirin was shown to increase the abundance of *Prevotella*, *Akkermansia*, and *Ruminococcaceae* species while decreasing the levels of *Bacteroides*, *Parabacteroides* and *Dorea* species, that have collectively been associated with reduced CRC risk [93]. Another in vivo experiment with germ-free and conventionalized mice also demonstrated that aspirin was able to increase the probiotic bacteria *Bifidobacterium* and *Lactobacillus*, suggestive of a role in CRC prevention. However, this study also demonstrated that the aerobic microbe *Lysinibacillus sphaericus* was capable of degrading aspirin in the lumen; supplementation with antibiotics prevented such microbe-mediated aspirin degradation. Their study also pointed out the ability of aspirin to inhibit tumor formation in germ-free mice, raising the possibility that aspirin may prevent CRC through a microbiome-independent mechanism, and suggested that this may involve inhibition of COX-2 and PGE2 synthesis [94]. This raises the possibility that aspirin may exert anti-cancer effects independent of the formation of metabolites. A role for aspirin in increasing the beneficial bacteria which may contribute to human gut health and cancer prevention should be explored in future investigations. Under normal conditions, a portion of aspirin taken orally may be left unabsorbed, and therefore has the potential to act directly on colorectal tissues. However, aspirin may also be subjected to metabolism, generating 2,3-DHBA and 2,5-DHBA, that in turn act on the colorectal tissues to prevent cancer. As 2,5-DHBA and 2,3-DHBA have been shown to inhibit cancer cell growth [45,54], it would be interesting to determine if these aspirin metabolites can potentiate the cancer preventive properties of aspirin when administered to germ-free mice. Such studies should validate the significance of both intact aspirin and its metabolites collectively contributing to CRC prevention.

## 5. Perspective and Future Studies

Cancer is a genetic disease which arises due to mutations in protooncogenes and tumor suppressor genes whose imbalances in expression lead to uncontrolled cell proliferation. Although traditionally viewed as a disorder of cell proliferation, recent evidence suggests that cancer should also be considered as a metabolic disease owing to reprogramming of the metabolic pathways to meet increased cellular demands for energy and growth [95]. Therefore, effective preventive measures for cancer require identification of compounds that not only target proteins that regulate cell proliferation, but also those that affect cell metabolism. In this regard, aspirin holds great promise for the prevention of cancer; however, despite considerable efforts in identifying the mechanism of cancer prevention by aspirin, a consensus has not yet been reached. It appears that aspirin and its primary metabolite salicylic acid affect many enzymes, proteins, transcription factors, and signaling pathways involved in cell proliferation and cancer development; thus, the wide range of targets recognized for aspirin has made the identification of its primary target difficult.

Broadly speaking, researchers have classified the known mechanisms for aspirin’s cancer prevention under two categories—COX-dependent and COX-independent pathways. As mentioned above, the platelet hypothesis (COX-dependent pathway) proposes sequential inhibition of COX-1 enzyme activity in platelets and COX-2 expression in nucleated cells of the colonic mucosa [6,29]. While it is an interesting and attractive hypothesis, it has not been conclusively proven. One major issue is that the hypothesis proposed is indirect and requires the involvement of multiple steps and factors to execute the observed anti-cancer effects. Additionally, the primary target (platelet COX-1) and subsequent downstream effector (epithelial/COX-2) are in different cell types. The platelet hypothesis also does not explain the preferential protective effects of aspirin against CRC as compared to other cancers. If platelet COX-1 inhibition is primarily responsible for cancer prevention following its absorption into circulation, it is reasonable to expect that aspirin should equally be effective against all cancers, which is not the case. Moreover, according to this hypothesis, the chemopreventive effects of aspirin are applicable against CRC that is developed after mucosal injury led platelet activation; it does not explain how low-dose aspirin works against cancers due to sporadic mutations where platelets may not be involved. This hypothesis was proposed to accommodate the inability of low-dose aspirin to directly inhibit COX-2, an important COX isoform implicated in the development of many cancers. Since low-dose aspirin was as effective as higher doses in preventing CRC, and the only known effect of low dose aspirin was COX-1 inhibition in platelets, the proponents of the platelet hypothesis opined that the inhibition of COX-1 in platelets may be central to the prevention of tumorigenesis. It is important to note that although COX-2 expression is implicated in the development of CRC, aspirin has been shown to be effective in inhibiting cancer cell growth even in cells that do not overexpress COX-2 [96]. Additionally, it has also been observed that some CRC cell lines do not produce COX-2 or they express an inactive form of COX-2 [97]. Another report also showed that cancers affecting the proximal colon, that generally do not over-express COX-2 [98], were more responsive to aspirin use than those cancers of the distal colon [7]. These observations raise the possibility that alternate pathways, independent of COX inhibition, may also play a substantial role in aspirin’s chemopreventive effects. However, prevention of platelet aggregation by aspirin may be important in curtailing the metastatic spread of cancer cells as platelet aggregation and activation has previously been implicated in cancer cell growth, cancer cell survival in circulation, protection from immune destruction and angiogenesis at sites of metastasis [99,100,101,102].

Aspirin’s preferential effects against CRC compared to cancers of other tissues raises the possibility that it may act directly on colorectal tissues before its absorption into circulation. Such an effect requires substantial concentrations of aspirin in the intestinal milieu which we believe is achievable. Since the bioavailability of aspirin is ~50% [103] and there is significant hydrolysis of aspirin occurring in the intestine [32], colorectal tissues are likely to be exposed to millimolar concentrations of aspirin and salicylic acid. This will be particularly true following consumption of enteric-coated tablets as its absorption is significantly lower than regular aspirin [33,104] making it more available in the lumen of the intestine. In such an environment, both aspirin and salicylic acid can target specific proteins in the colonic cells to modulate their function and bring about changes required for effective chemoprevention. In this context, intact aspirin has the potential to target proteins through acetylation, and along with salicylic acid, may also target specific transcription factors such as NF-κB or c-myc in colorectal tissues. Alternatively, other signaling pathways like Wnt/β-catenin or Akt/mTOR pathways may also be regulated as described above by aspirin and salicylic acid under these conditions.

Equally important are also the role of aspirin metabolites 2,3-DHBA and 2,5-DHBA in its chemopreventive effects. Both DHBAs have been demonstrated to inhibit CDK enzyme activity and colony formation in cancer cells, although 2,5-DHBA was more potent when compared to 2,3-DHBA [45,46]. In view of the suggestion that intestinal bacteria are capable of degrading aspirin and salicylic acid to DHBAs, their contribution to the chemopreventive effect of aspirin should also be considered. Except for a few reports, a role for HBAs in cancer prevention has not been investigated in depth [45,46,54]. Exploring the role played by HBAs in aspirin’s cancer prevention should take precedence in future investigations as they are also generated following degradation of flavonoids [105,106,107,108], which are known to prevent cancer. Interestingly, the aspirin metabolite 2,5-DHBA is also produced from the degradation of flavonoids in the human gut, and this has been detected both in the plasma and urine samples from test subjects [60,62]. In addition, there is plenty of evidence in the literature that increased consumption of fruits and vegetables, that are rich in HBAs, is inversely correlated to the occurrence of cancer [59,60,61,62]; an interesting target proposed for HBAs are CDKs present in colonic epithelial cells [44,45,46,109]. Some reports also indicate that the phenolic acid content in the human gut can collectively reach millimolar concentrations [62,110]. If one considers HBAs as possible common mediators of CRC prevention, it provides the simplest explanation through which all three compounds (aspirin, flavonoids and a diet rich in HBAs) can exert anti-cancer effects. Thus, the metabolite hypothesis borne out of the finding that HBAs inhibit cancer cell growth [44,45,46,109] needs to be explored in future research with regards to its targets and mechanisms of action.

The literature is immense on the mechanisms of cancer prevention by aspirin, yet they are unclear and complex even after two decades of research. Going back to the question—“how does low-dose aspirin preferentially act to protect against CRC?”—neither a consensus has been reached with regard to the mechanisms responsible for its cancer preventive actions nor has a single target been defined as solely responsible for its protective effect. This preferential preventive effect may mainly be attributed to the fact that colorectal tissues are the first to get exposed to aspirin/salicylic acid or its metabolites following oral consumption. In our view, aspirin or its metabolites acting locally on colorectal tissues to target specific proteins appear to be the most simplistic explanation as compared to aspirin targeting platelet COX-1 in the circulation, leading to prevention of tumorigenesis. However, aspirin through inhibition of platelet COX-1 may play a role in preventing cancer metastasis, which is also important in the overall anti-cancer effects of aspirin. We believe that in view of aspirin’s reported ability to prevent CRC, efforts should be put-forth to resolve this important dilemma. In this regard, the recently described metabolite hypothesis along with other targets of aspirin, acting directly on colorectal tissues should be further explored. We hope that a resolution would be reached in the near future on aspirin’s mode of action against CRC through scientific research and open debate.

## Abbreviations

2,3-DHBA	2,3-dihydroxybenzoic acid
2,5-DHBA	2,5-dihydroxybenzoic acid
5-ASA	5-aminosalicylic acid
AMPK	Adenosine monophosphate activated protein kinase
AMP	Adenosine MonoPhosphate
APC	Adenomatous polyposis coli
ATP	Adenosine TriPhosphate
CDK	Cyclin Dependent Kinase
COX	Cyclooxygenase
CYP450	Cytochrome P450
CRC	Colorectal cancer
EMT	Epithelial-mesenchymal transition
FGF	Fibroblast growth factor
G6PD	glucose-6-phosphate dehydrogenase
GI	Gastrointestinal
HBA	Hydroxybenzoic acid
IKK	IκB Kinase
mTOR	Mechanistic target of rapamycin
NF-κB	Nuclear factor -κB
NSAID	Non-steroidal anti-inflammatory drug
ODC	Ornithine decarboxylase
PDGF	Platelet-derived growth factor
PG	Prostaglandin
PGI2	Prostacyclin
SSAT	Spermidine/spermine N1-acetyltransferase
TXA-2	Thromboxane A2
USPSTF	United States Preventive Services Task Force
